# The impact of social vulnerability index on survival following autologous stem cell transplant for multiple myeloma

**DOI:** 10.1038/s41409-024-02200-x

**Published:** 2024-01-18

**Authors:** Kiarash Salafian, Christine Mazimba, Leonid Volodin, Indumathy Varadarajan, Asal Pilehvari, Wen You, Ziyad O. Knio, Karen Ballen

**Affiliations:** 1 Department of Medicine, University of Virginia Health, Charlottesville, VA USA; 2 Division of Hematology/Oncology, University of Virginia Health, Charlottesville, VA USA; 3 Department of Public Health Sciences, University of Virginia, and University of Virginia Comprehensive Cancer Center, Charlottesville, VA USA; 4 Department of Anesthesiology, University of Virginia Health, Charlottesville, VA USA

**Keywords:** Myeloma, Risk factors, Public health

## Abstract

Autologous hematopoietic stem cell transplantation (ASCT) is the standard of care for eligible patients with multiple myeloma (MM) to prolong progression-free survival (PFS). While several factors affect survival following ASCT, the impact of social determinants of health such as the CDC Social Vulnerability Index (SVI) is not well documented. This single-center retrospective analysis evaluated the impact of SVI on PFS following ASCT in MM patients. 225 patients with MM who underwent ASCT participated, with 51% transplanted in the last 5 years. At 5 years post-transplant, 55 (50%) achieved PFS and 66 (60%) remained alive. Higher SVI values were significantly associated with lower odds of PFS (OR = 0.521, *p* < 0.01, 95% CI [0.41, 0.66]) and OS (OR = 0.592, *p* < 0.01, 95% CI [0.46, 0.76]) post-transplant. Greater vulnerability scores in the socioeconomic status (OR = 0.890; 95% CI: [0.82, 0.96]), household characteristics (OR = 0.912; 95% CI: [0.87, 0.95]), and racial and ethnic minority status (OR = 0.854; 95% CI: [0.81, 0.90]) themes significantly worsened the odds of PFS. These results suggest high SVI areas may need more resources to achieve optimal PFS and OS. Future studies will focus on addressing factors within the socioeconomic status, household characteristics, and racial and ethnic minority subthemes, as these have a more pronounced effect on PFS.

## Introduction

The prognosis of patients with multiple myeloma (MM) has significantly improved with the introduction of novel agents. High-dose chemotherapy with an autologous hematopoietic stem cell transplant (ASCT) continues to be the standard of care for eligible patients with MM [1]. ASCT has been proven to prolong progression-free survival (PFS) compared to combination chemotherapy alone and the addition of maintenance therapy after ASCT has further improved PFS [1–5].

**Fig. 1:**
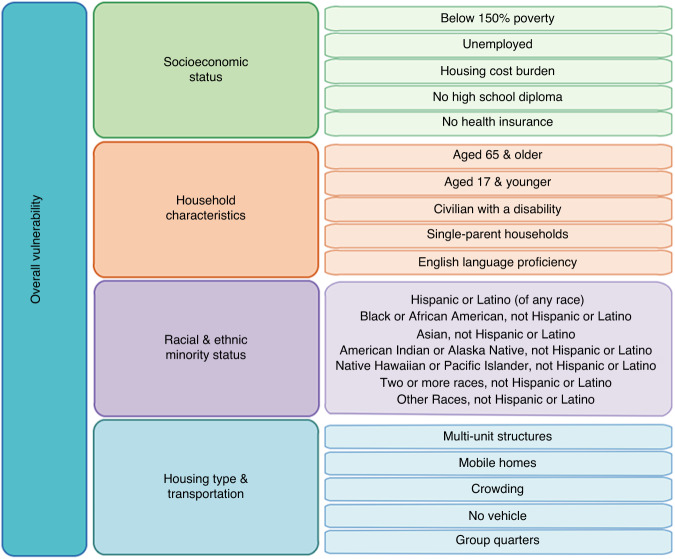
SVI and its four subthemes comprised of 16 social factors. Source: CDC/ATSDR. Note: Use of figure does not imply endorsement by CDC/ATSDR.

While several factors affect survival following ASCT, the impact of social determinants of health, especially the local level social vulnerability, on survival in this patient population is not well documented. This study used the Centers for Disease Control and Prevention (CDC) Social Vulnerability Index (SVI) to capture local levels of social vulnerability. Composed of 16 social factors, SVI is a constantly evolving measure of a community’s ability to respond to hazardous events and is traditionally used to help public health officials identify populations in most need of support [6]. As outlined in Fig. 1, SVI is comprised of 16 social factors grouped into four related themes (socioeconomic status; household characteristics; racial and ethnic minority status; and housing type and transportation). These individual components and themes comprise a composite SVI score that reflects a community’s overall vulnerability ranking [6]. Areas with higher SVI values are at a higher risk during public health emergencies and are considered more socially vulnerable.

SVI has traditionally been used for natural disaster outreach, but recently has been studied in cancer patients [7, 8]. Previous studies from our group examining acute myeloid leukemia (AML) patients have shown that patients living in high SVI areas are less likely to undergo allogeneic hematopoietic cell transplantation (HCT); other centers have documented worse survival outcomes after HCT for AML [9, 10]. However, to our knowledge, the impact of local level social vulnerability has not been studied after MM ASCT.

In this paper, we seek to understand the impact of SVI on outcomes in patients with MM undergoing ASCT.

## Materials and methods

### Patients

A single-center retrospective study of MM patients who underwent ASCT between January 1, 2012, and December 31, 2020, was performed. Patients were excluded if they underwent ASCT without a diagnosis of MM, had a diagnosis of amyloidosis, or if their county of residence was not located in the Commonwealth of Virginia. Patients were followed from the time of diagnosis to 5 years post-transplant, if applicable. The study protocol was approved by the Institutional Review Board of the University of Virginia. Informed consent of patients was waived, given the retrospective nature of the study.

### Variables

Data collected included date of birth, sex (defined as male or female), race, ZIP code, SVI, primary insurance payor (public vs. private), geographic location (urban vs. rural), date of MM diagnosis, cytogenetic data, risk stratification of disease, post-transplant maintenance therapy received, date of disease progression, last follow up date, and date of death, if applicable. Race was collected based on self-reported patient data documented in the electronic medical record and was defined as Non-Hispanic White, Black, Asian, and Other (including Latinx). Mayo Stratification for Myeloma and Risk-Adapted Therapy (mSMART) 3.0 criteria was used to stratify disease risk and the International Myeloma Working Group (IMWG) Uniform Response Criteria for Multiple Myeloma was used to determine disease response [11, 12]. Specifically, response was classified as progressive disease if there was an increase of >25% from lowest response value in any one or more of the following:

1. Serum M-component (absolute increase must be >0.5 g/dL) and/or
2. Urine M-component (absolute increase must be >200 mg/24 h) and/or
3. Only in patients without measurable serum and urine M-protein levels: the difference between involved and uninvolved free light chain (FLC) levels. The increase must be >10 mg/dL.
4. Bone marrow plasma cell percentage: the absolute percentage must be >10%
5. Definite development of new bone lesions or soft tissue plasmacytomas or definite increase in the size of existing bone lesions or soft tissue plasmacytomas
6. Development of hypercalcemia (corrected serum calcium >11.5 mg/DL or 2.65 mmol/L) that can be attributed solely to the plasma cell proliferative disorder [12].

The geographic variable in our data set was zip code. Patients’ self-reported ZIP codes, documented in the electronic medical record, were used to identify the county in Virginia they resided in using the U.S. Department of Housing and Urban Development (HUD) United States Postal Service (USPS) ZIP Code Crosswalk Files. ZIP codes (and their corresponding counties) were classified as urban or rural using the rural-urban continuum codes provided by the U.S. Department of Agriculture [13].

The primary independent variable was SVI. SVI was obtained by corresponding patients’ counties to their respective SVI values using the CDC and Agency for Toxic Substances and Disease Registry (ATSDR) SVI database, freely available online. SVI is a composite score that indicates the relative vulnerability of every U.S. Census tract and county and is derived from U.S. census tract data [6]. SVI is a percentile ranking value ranging from 0.00 to 1.00, with higher values indicating greater vulnerability. The composite SVI score is made up of 16 variables which comprise four main themes: socioeconomic status; household characteristics; racial and ethnic minority status; and housing type and transportation (Fig. 1). The four SVI themes are also given percentile rankings by summing the percentiles for each variable comprising the respective theme [6].

### Statistical analysis

We conducted statistical tests to compare the characteristics of patients (including demographics and clinical characteristics) across the levels of SVI, which were categorized as low SVI and high SVI counties of residence. The classification of SVI into low and high categories was determined by the median value of SVI distribution within our study population. These comparisons were performed for the overall sample, as well as subsamples of patients who were progression-free and patients who survived. Continuous variables were analyzed using t-tests, while chi-squared tests were conducted for categorical variables.

Logistic regression models were used to examine the association between SVI and the probability of both PFS as the primary outcome, and overall survival (OS) as the secondary outcome. *P* < 0.05 were deemed to be statistically significant. Kaplan-Meier (KM) curves were generated to illustrate the differences in PFS and OS probabilities between low and high SVI groups (defined by median of SVI distribution). The KM curve analysis, complemented by the logrank test, was adjusted for patients’ clinical characteristics, including risk stratification and maintenance therapy. Additionally, logistic regression models were utilized to evaluate the association between the four SVI themes (one at a time) and probability of PFS and OS outcomes.

## Results

### Patients

A total of 225 patients met inclusion criteria and were evaluated in this study; baseline characteristics are summarized in Table 1. The majority (*n* = 115, 51.1%) of patients underwent transplant in the last 5 years; 110 patients (48.9%) were evaluable at the study endpoint of 5 years post-transplant. Patients were on average 61.9 years old. The majority (*n* = 161, 71.6%) of patients were Non-Hispanic White, with the next largest racial group being Black (*n* = 48, 21.3%). Most patients were male (*n* = 145, 64.4%) and had public insurance payors (*n* = 133, 59.6%). The majority of patients lived in urban areas (*n* = 147, 65.3%); thirty-five percent lived in rural areas, reflecting the referral population for our cancer center, which serves large areas of Appalachia. Nearly all patients received post-transplant therapy (*n* = 209, 95%) and over one-third had high-risk disease (*n* = 77, 35.2%).

**Table 1:** Patient characteristics on overall sample and by subsamples based on SVI.

	Total	Low SVI^a^	High SVI		*P* value	
Variable	No. (%)	No. (%)	No. (%)			
Age (mean (SD))	61.9 (8.3)	62.1 (8.4)	61.7 (8.4)		0.72	
Gender					0.08	
Female	80 (35.6)	46 (41.1)	34 (30.1)			
Male	145 (64.4)	66 (58.9)	79 (69.9)			
Race/Ethnicity					0.14	
Black	48 (21.3)	19 (17)	29 (25.7)			
Asian	2 (0.9)	2 (1.8)	0 (0)			
Other	14 (6.2)	9 (8)	5 (4.4)			
Non-Hispanic White	161 (71.6)	82 (73.2)	79 (69.9)			
Health Insurance					0.25	
Private	90 (40.4)	49 (44.1)	41 (36.6)			
Public	133 (59.6)	62 (55.9)	71 (63.4)			
Disease Risk					0.85	
Standard	111 (50.7)	56 (51.9)	55 (49.5)			
Intermediate	21 (9.6)	9 (8.3)	12 (10.8)			
High	77 (35.2)	39 (36.1)	38 (34.2)			
Unknown	10 (4.6)	4 (3.7)	6 (5.4)			
Post-Transplant Therapy					0.52	
None	11 (5)	6 (5.5)	5 (4.5)			
Lenalidomide	100 (45.5)	55 (50)	45 (40.9)			
Bortezomib	62 (28.2)	26 (23.6)	36 (32.7)			
Other	8 (3.6)	3 (2.7)	5 (4.5)			
Multiple	39 (17.7)	20 (18.2)	19 (17.3)			
Geography					<0.001	
Rural	78 (34.7)	16 (14.3)	62 (54.9)			
Urban	147 (65.3)	96 (85.7)	51 (45.1)			
Total	225 (100)	112 (100)	113 (100)			

^a^Low (High) SVI pertains to counties where the SVI level falls below (above) the median SVI value (0.36) within the study sample.

### Social vulnerability index

The median composite SVI of our study population was 0.36, with 113 patients being classified as living in high SVI counties and 112 living in low SVI counties (SVI above and below the median, respectively). The results presented in Table 1 demonstrate differences between the two groups. Analysis of the urban-rural status revealed a substantial difference, as 14.3% (*n* = 16) of patients in low SVI areas resided in rural areas, while 54.9% (*n* = 62) of patients in high SVI areas lived in rural areas (*p* < 0.001). There were no differences in race/ethnicity, gender, insurance type, or disease risk between patients living in low SVI and high SVI areas.

## Regression analysis

### Progression free survival

Tables 2 and 3 summarize the findings of a logistic regression analysis, investigating how SVI influences the probability of PFS and OS while also considering other factors, such as patient demographics, residential features, and clinical characteristics. Notably, higher SVI levels were significantly associated with lower odds of PFS (OR = 0.52, 95% CI: [0.41, 0.66]). Several additional factors were found to have a significant association with the likelihood of PFS. Patients who lived in urban areas had improved PFS (OR = 1.22, 95% CI: [1.13, 1.32]) while female patients (OR = 0.77, 95% CI: [0.72, 0.83]), Non-Hispanic White patients (OR = 0.63, 95% CI: [0.61, 0.67]), and patients with private insurance payors (OR = 0.80, 95% CI: [0.76, 0.85]) were all significantly associated with a lower odds of PFS. As expected, patients who had intermediate (OR = 0.63, 95% CI: [0.55, 0.73]) or high-risk disease (OR = 0.39, 95% CI: [0.34, 0.44]) had lower odds of PFS.

**Table 2:** Adjusted odds ratio depicting factors impacting progression free survival.

Variable	PFS [95% CI]	*P*
Age	1.01 [1.00, 1.01]	<0.01
Race/Ethnicity		
Non-Hispanic White	0.64 [0.61, 0.67]	<0.01
Other Race/Ethnicity	1.00 (Referent)	---
Health Insurance		
Private	0.80 [0.76, 0.85]	<0.01
Public	1.00 (Referent)	---
County Features		
Urban	1.22 [1.13, 1.32]	<0.01
Rural	1.00 (Referent)	
Overall SVI^a^	0.52 [0.41, 0.66]	<0.01
Disease Risk		
Standard	1.00 (Referent)	---
High	0.39 [0.34, 0.44]	<0.01

^a^SVI index is continuous with higher values indicating higher levels of vulnerability.

**Table 3:** Adjusted odds ratio depicting factors impacting overall survival.

Variable	OS [95% CI]	*P*
Age	1.00 [0.99, 1.00]	NS
Race/Ethnicity		
Non-Hispanic White	0.92 [0.87, 0.97]	<0.01
Other Race/Ethnicity	1.00 (Referent)	---
Health Insurance		
Private	0.76 [0.71, 0.81]	<0.01
Public	1.00 (Referent)	---
County Features		
Urban	1.76 [1.61,1.92]	<0.01
Rural	1.00 (Referent)	
Overall SVI^a^	0.59 [0.46, 0.76]	<0.01
Disease Risk		
Standard	1.00 (Referent)	---
High	0.69 [0.60, 0.78]	<0.01

^a^SVI index is continuous with higher values indicating higher levels of vulnerability.

**Fig. 2:**
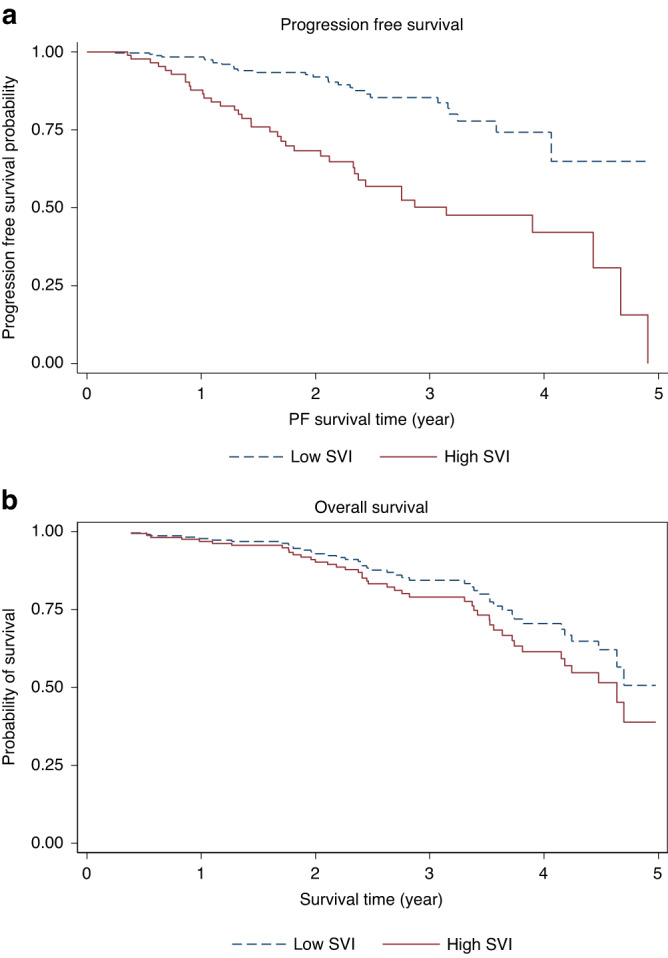
Effect of SVI on PFS (a) and OS (b). **a** Kaplan-Meier curve depicting Progression Free Survival of High vs. Low SVI Groups. Note: The KM curve was adjusted for risk stratification and post-transplant therapy. Logrank test *p* = 0.01. **b** Kaplan-Meier curve depicting Overall Survival of High vs. Low SVI Groups. Note: The KM curve was adjusted for risk stratification and post-transplant therapy. Logrank test *p* = 0.01.

In total, 55 patients (50%) showed no progression at the study endpoint of 5 years post-transplant. Survival analysis revealed that patients living in low SVI areas had a 2-year PFS of 84.7% and 3-year PFS of 78.6%, while patients living in high SVI areas had a 2-year PFS of 76.9% and 3-year PFS of 69.0%. The KM curve in Fig. 2a presents PFS by low versus high SVI. As shown in Fig. 2, the probability of PFS is higher in areas with low SVI status. Moreover, as the number of days post-transplant increases, the difference in PFS increases. The logrank test results show that there is a significant difference between PFS for patients stratified by SVI (*p* = 0.01).

### Overall survival

As shown in Table 3, higher levels of SVI were associated with a decreased odds of OS (OR = 0.59, 95% CI: [0.46, 0.76]). Similar to PFS, patients living in urban areas had a greater likelihood of survival (OR = 1.76, 95% CI: [1.61, 1.92]). Non-Hispanic White patients (OR = 0.92, 95% CI: [0.87, 0.97]), patients with private insurance (OR = 0.76, 95% CI: [0.71, 0.81]), and those with high-risk disease had lower odds of OS (OR = 0.69, 95% CI: [0.60, 0.78]).

In total, OS was 60% (66 patients) at 5 years post-transplant. Survival analysis showed patients living in low SVI areas had a 2-year OS of 93.7% and 3-year OS of 88%, while patients living in high SVI areas had a 2-year OS of 90.3% and 3-year OS of 84%. As shown by the KM curve in Fig. 2b, the probability of OS is higher in areas with low SVI levels. The logrank test results show that there is a significant difference between OS for patients stratified by levels of SVI (*p* = 0.01).

### Social vulnerability index and its subanalyses

When examining the four themes of the SVI, the majority of the themes showed decreased odds of both PFS and OS, as shown in Table 4. Furthermore, higher vulnerabilities in the socioeconomic theme (OR = 0.89, 95% CI: [0.82, 0.96]), household composition theme (OR = 0.91, 95% CI: [0.87, 0.95]), and racial and ethnic minority theme (OR = 0.85, 95% CI: [0.81, 0.90]) significantly worsened the odds of PFS when considered as continuous values. However, no statistically significant impact on the odds of PFS was found for the housing type and transportation theme.

**Table 4:** Odds ratio estimation of the impact of different SVI subthemes on PFS and OS.

	Socioeconomic Status^a^	Household Composition and Disability^a^	Racial and Ethnic Minority Status^a^	Housing Type and Transportation^a^
PFS [95% CI]	0.89 [0.82, 0.96]^b^	0.91 [0.87, 0.95]^c^	0.85 [0.81, 0.90]^c^	0.98 [0.91, 1.05]
OS [95% CI]	0.92 [0.85, 0.99]^b^	0.87 [0.83, 0.91]^c^	1.01 [0.95, 1.07]	1.01 [0.95, 1.07]

^a^SVI subthemes are continuous with higher values indicating higher levels of vulnerability.

^b^Significant at the 5% level.

^c^Significant at the 1% level.

Regarding OS, increased vulnerabilities in the socioeconomic theme (OR = 0.92, 95% CI: [0.85, 0.99]) and household composition theme (OR = 0.87, 95% CI: [0.83, 0.91]) lowers the odds of OS. On the other hand, no significant association was observed between the racial and ethnic minority status theme or the housing theme and OS.

## Discussion

To our knowledge, this is the first study investigating the impact of SVI on post-transplant outcomes for patients with MM. This study determined that patients living in high SVI counties who underwent ASCT had significantly lower odds of PFS and OS as compared to patients living in low SVI counties. Our SVI subanalyses further identified that the socioeconomic status, household characteristics, and racial and ethnic minority status themes were most strongly associated with poorer odds of PFS. However, each individual theme’s association with PFS was less pronounced than composite SVI, which suggests that the variables comprising SVI have a synergistic effect.

These results are consistent with literature studying individual social determinants of health and their associations with MM outcomes. In the present study, Non-Hispanic Whites had a significantly lower odds of PFS and OS in comparison to other racial/ethnic groups (Black, Asian, other). Saraf et al. conducted a retrospective analysis investigating outcomes of MM patients post-ASCT and demonstrated that Black patients had a significantly increased median event-free survival compared to non-Black patients (21 vs 12 months, *p* = 0.02) [14]. Ailawadhi et al. similarly found that Black patients had significantly longer median multiple MM specific survival (MSS) in comparison to Whites (5.4 years vs 4.5 years, respectively; p < 0.05) and comparable median MSS for Hispanics and Whites (4.9 vs 4.5 years, respectively; *p* = 0.41) [15]. More recently, Dong et al. found that Black patients had significantly longer 5-year OS than Non-Hispanic White patients, when they were treated similarly (absolute difference =3.8%, *p* = 0.003) [16]. It has been thought that this improved survival in Black patients with MM may be due to more biologically indolent disease subtypes present in this population [17].

Our study found that patients living in urban areas had a significantly higher odds of PFS compared to patients from rural areas, even in the modern era, consistent with previous studies [18, 19]. Our cancer center has a uniquely high rural catchment area, allowing us to study rural/urban differences. Previously, Rao et al. concluded that patients from rural areas who received ASCT had a higher relative risk of death (RR = 1.18, p = 0.016) compared to patients from urban areas [18]. Survival at 1 year (73% vs 78%, *p* < 0.04) and 5 years (48% vs 54%; *p* = 0.12) were also lower for patients from rural areas versus urban areas, respectively [18]. This disparate outcome is believed to be due in part to rural patients having to travel greater distances to access quality health care, delay in seeking treatments, and modifiable risk factors such as tobacco use and obesity [20, 21].

The ATSDR in conjunction with the CDC, created the CDC/ATSDR SVI to assist public health officials and emergency response planners identify communities that will most likely need support, during, and after a hazardous event [6]. However, SVI is being increasingly utilized in medical health outcomes research, particularly in the field of surgery [22]. For example, Azap et al. demonstrated that patients with high SVI had decreased odds (OR = 0.89, 95% CI 0.82–0.97) of achieving superior outcomes after pancreatic surgery [23]. Hyer et al. similarly reported that patients from high SVI areas undergoing common oncologic surgical procedures had a higher probability of 90-day mortality [24]. As a result, SVI is becoming increasingly recognized as helpful in government allocation of resources to cancer patients in hopes of reducing excess cancer morbidity and mortality in specific communities [25].

SVI is not the only measure of community/neighborhood level vulnerability as other indices have been studied in medical health outcomes research [26–30]. Carmichael and colleagues (2020) determined that SVI performed similarly to other indices of neighborhood vulnerability (Area Deprivation Index, Community Needs Index (CNI), and Distressed Communities Index (DCI)) in predicting emergent surgical presentations but had several advantages [31]. Unlike other measures of neighborhood vulnerability, SVI is maintained by the CDC/ATSDR (as opposed to a private organization or university) and is frequently updated. SVI is additionally available at the census tract level, offering a more granular view of a patient’s social vulnerability. In contrast, CNI and DCI capture data at the ZIP code level.

The current study is additionally strengthened by the analysis of the four subthemes of SVI and their impact on a patient’s probability of achieving PFS and OS post-ASCT. We demonstrated that patients living in areas with higher vulnerability scores in the socioeconomic status, household characteristics, and racial and ethnic minority status subthemes had significantly lower odds of PFS post-ASCT. Higher vulnerability values in the socioeconomic and household characteristics subthemes were also associated with lower OS post-ASCT. Interestingly, increases in the housing type and transportation subtheme (which encompasses transportation, housing structure, etc.) were not associated with lower odds of PFS or OS. This may be due to our institution’s ability to provide subsidized housing and transportation.

While data are scarce investigating social vulnerability and outcomes following HCT, Bhandari et al. studied outcomes in patients who underwent allogeneic HCT [10]. Their group similarly found that the highest values in the socioeconomic status, household characteristics, and racial and ethnic minority status subthemes were associated with a higher odds of 1-year non-relapse mortality [10].

Our study has several limitations. We performed a single-institution retrospective study investigating patients who live in the Commonwealth of Virginia. While our findings may be unique to the demographics of Virginia, Virginia demographics are similar to the US (Virginia 60% White, 19% Black, 11% Hispanic, 7% Asian and US 62% White, 12% Black, 19% Hispanic, 6% Asian) [32]. An important feature of our Cancer Center is to increase access to cancer care for rural populations; hence, 35% of our patients were from rural areas, including Appalachia. Appalachia is associated with a rural population, and a high prevalence of poverty, obesity, and substance use, which may explain the high SVI seen in rural areas in our study [33]. In contrast, urban areas in our study include wealthy suburbs of Washington D.C., which likely accounts for the lower SVI in these counties. Also, our SVI scores were relative values at the state level and thus, these values will change when compared to counties across states. An additional limitation is our limited sample size and study population consisting of predominantly Non-Hispanic White patients. Furthermore, since SVI was studied at the county level it may not reflect patient-specific risks. Lastly, because SVI is a composite score, it can be difficult to discern which of the discrete individual factors have the most profound effect on one’s PFS or OS. However, our study serves as a launching point for future investigations in answering this as we have shown which of the 4 SVI subthemes have a significant association with PFS and OS post-ASCT.

In conclusion, this study presents important results in the field of HCT. Our institution utilized county-level SVI as a comprehensive measure of patients’ vulnerability and determined that patients living in areas of high social vulnerability have worse odds of PFS and OS post-ASCT. The subtheme analysis also reveals key variables that future studies can highlight. Specifically, the study results illustrated that the socioeconomic status, household characteristics, and racial and ethnic minority status themes were significantly associated with lower odds of PFS. Therefore, further research will be focused on these subthemes and the variables that make up these groups. For example, for patients living in areas with higher household vulnerability, we would plan to investigate the impact of assistance with family/caretaker support in hopes that this may improve survival outcomes. This study highlights the importance of social vulnerability in post-transplant outcomes and provides a framework for future interventions to improve post-transplant outcomes.

### Supplementary information

Supplementary Table 1

## Data Availability

The data that support the findings of this study are available from the corresponding author upon reasonable request.

## Code availability

The code associated with this project is available upon request. Please contact the corresponding author for access to the code.
