# Supplementary material for: The impact of social vulnerability index on survival following autologous stem cell transplant for multiple myeloma
Source: Bone Marrow Transplant. 2024 Jan 18;59(4):459–65. doi: 10.1038/s41409-024-02200-x (PMC10994832; doi:10.1038/s41409-024-02200-x)
Supplement: Supplementary file 1 — Supplementary Table 1 [file 41409_2024_2200_MOESM1_ESM.pdf]

Supplementary Figures:

Supplementary Table 1: Summary Statistics of Patient Characteristics by Social Vulnerability Index (SVI) and Outcomes (PFS and OS)

Table 2: Summary Statistics of Patient Characteristics by Social Vulnerability Index (SVI) and Outcomes (PFS and OS)

	Overall sample							Progression-free sample					Survived sample				
	Low SVI		High SVI		Total			Low SVI		High SVI			Low SVI		High SVI		
Variable	No.	Col %	No.	Col %	No.	Col %	P-value [a]	No.	col %	No.	col %	P-value [a]	No.	col %	No.	col %	p-value [a]
**Age (mean (SD))**	62.1 (8.4)		61.7 (8.4)		61.9 (8.3)		0.72	62.2 (8.4)		62.1 (8.1)		0.97	61.4 (8.3)		62.5 (8.7)		0.39
**Gender**							0.08					0.03					0.02
Female	46	41.1	34	30.1	80	35.6		34	42.5	18	25.4		41	45.6	23	28.4	
Male	66	58.9	79	69.9	145	64.4		46	57.5	53	74.6		49	54.4	58	71.6	
Total	112	100	113	100	225	100		80	100	71	100		90	100	81	100	
**Race/Ethnicity**							0.14					0.48					0.39
Black	19	17	29	25.7	48	21.3		18	22.5	16	22.5		18	20	19	23.5	
Asian	2	1.8	0	0	2	0.9		2	2.5	0	0		2	2.2	0	0	
Other	9	8	5	4.4	14	6.2		7	8.8	4	5.6		8	8.9	4	4.9	
Non-Hispanic White	82	73.2	79	69.9	161	71.6		53	66.2	51	71.8		62	68.9	58	71.6	
Total	112	100	113	100	225	100		80	100	71	100		90	100	81	100	
**Health Insurance**							0.25					0.16					0.18
Private	49	44.1	41	36.6	90	40.4		36	45	24	33.8		39	43.8	27	33.8	
Public	62	55.9	71	63.4	133	59.6		44	55	47	66.2		50	56.2	53	66.2	
Total	111	100	112	100	223	100		80	100	71	100		89	100	80	100	
**Disease risk**							0.85					0.63					0.85
Standard	56	51.9	55	49.5	111	50.7		42	54.5	41	59.4		45	51.7	43	53.8	
Intermediate	9	8.3	12	10.8	21	9.6		6	7.8	8	11.6		8	9.2	10	12.5	
High	39	36.1	38	34.2	77	35.2		26	33.8	17	24.6		31	35.6	25	31.2	
Unknown	4	3.7	6	5.4	10	4.6		3	3.9	3	4.3		3	3.4	2	2.5	
Total	108	100	111	100	219	100		77	100	69	100		87	100	80	100	
**Post-Transplant Therapy**							0.52					0.24					0.31
None	6	5.5	5	4.5	11	5		4	5.1	1	1.5		3	3.3	0	0	
Lenalidomide	55	50	45	40.9	100	45.5		48	61.5	35	51.5		46	51.1	36	45	
Bortezomib	26	23.6	36	32.7	62	28.2		16	20.5	23	33.8		21	23.3	27	33.8	
Other	3	2.7	5	4.5	8	3.6		3	3.8	1	1.5		3	3.3	3	3.8	
Multiple	20	18.2	19	17.3	39	17.7		7	9	8	11.8		17	18.9	14	17.5	

Total	110	100	110	100	220	100		78	100	68	100		90	100	80	100	
**Rurality**							<0.001										<0.001
Rural	16	14.3	62	54.9	78	34.7		12	15	35	49.3		15	16.7	38	46.9	
Urban	96	85.7	51	45.1	147	65.3		68	85	36	50.7	<0.001	75	83.3	43	53.1	
Total	112	100	113	100	225	100		80	100	71	100		90	100	81	100	

Note: [a] Continuous variables were analyzed using t-tests, while chi-squared tests were employed for categorical variables.
